# Development and validation of functional kompetitive allele-specific PCR markers for herbicide resistance in *Brassica napus*

**DOI:** 10.3389/fpls.2023.1213476

**Published:** 2023-11-23

**Authors:** Jianghua Shi, Huasheng Yu, Ying Fu, Tanliu Wang, Yaofeng Zhang, Jixiang Huang, Sujuan Li, Tao Zheng, Xiyuan Ni, Jianyi Zhao

**Affiliations:** ^1^ Institute of Crop and Nuclear Technology Utilization, Zhejiang Academy of Agricultural Science, Hangzhou, China; ^2^ Central Laboratory, State Key Laboratory for Managing Biotic and Chemical Threats to the Quality and Safety of Agro-products, Zhejiang Academy of Agricultural Science, Hangzhou, China; ^3^ Institute of Biotechnology, Zhejiang Academy of Agricultural Science, Hangzhou, China

**Keywords:** KASP assay, herbicide resistance, SNPs, ALS genes, marker-assisted selection

## Abstract

Effective weed control in the field is essential for maintaining favorable growing conditions and rapeseed yields. Sulfonylurea herbicides are one kind of most widely used herbicides worldwide, which control weeds by inhibiting acetolactate synthase (ALS). Molecular markers have been designed from polymorphic sites within the sequences of ALS genes, aiding marker-assisted selection in breeding herbicide-resistant rapeseed cultivars. However, most of them are not breeder friendly and have relatively limited application due to higher costs and lower throughput in the breeding projects. The aims of this study were to develop high throughput kompetitive allele-specific PCR (KASP) assays for herbicide resistance. We first cloned and sequenced *BnALS1* and *BnALS3* genes from susceptible cultivars and resistant 5N (*als1als1/als3als3* double mutant). Sequence alignments of *BnALS1* and *BnALS3* genes for cultivars and 5N showed single nucleotide polymorphisms (SNPs) at positions 1676 and 1667 respectively. These two SNPs for *BnALS1* and *BnALS3* resulted in amino acid substitutions and were used to develop a KASP assay. These functional markers were validated in three distinct BC_1_F_2_ populations. The KASP assay developed in this study will be valuable for the high-throughput selection of elite materials with high herbicide resistance in rapeseed breeding programs.

## Highlights

- Developed KASP assays for *BnALS* genes are high throughput, low-cost, and capable of screening for herbicide-resistant alleles for marker-assisted selection.

## Introduction

Rapeseed (*Brassica napus* L., AACC) is one of the most important oil-producing crops worldwide, with an annual production of more than 28 million tons of vegetable oil globally (USDA ERS, 2021) and also provides important raw material for biofuel and other industrial products (Ohlrogge, 1994; Thelen and Ohlrogge, 2002). Weeds, especially the broad leaf cruciferous species, are well adapted to compete with rapeseed for sunlight, water, soil nutrients and physical space in the fields (Miki et al., 1990; Larue et al., 2019). Hence, weeds are a significant problem and greatly limit rapeseed yield. The development of herbicide-tolerant varieties is a high priority for varietal development and the most cost-effective tool to manage weeds (Tan et al., 2005; Green, 2014).

Acetolactate synthase (ALS) is the key enzyme for the biosynthesis of the branched chain amino acids, including valine, leucine, and isoleucine (Duggleby et al., 2008; Garcia et al., 2017). ALS has been proved to be the target site of several important herbicides, such as sulfonylurea (SU), imidazolinone (IMI), triazolopyrimidine (TP), pyrimidinyl-thiobenzoates (PTB) and sulfonyl- aminocarbonyl-triazolinone (SCT) (Yu and Powles, 2014). ALS harboring amino acid substitutions caused by gene editing or ethyl methane sulfonate (EMS) mutagenesis has been found to confer high resistance to sulfonylurea herbicides in crops including wheat, rapeseed and watermelon (Tian et al., 2018; Zhang et al., 2019; Guo et al., 2020). The genome information derived from *Brassica napus* cultivars Darmor-bzh and ZS11 shows that there are five copies in the *BnALS* gene family (Chalhoub et al., 2014; Sun et al., 2017). Of these, *BnALS1* and *BnALS3* are highly conserved, and constitutively expressed in all tissues (Wu et al., 2020). Thus, *BnALS1* and *BnALS3* are regarded to be essential *ALS* housekeeping genes and the ideal herbicide-resistance targets for genetic manipulation (Rutledge et al., 1991; Wu et al., 2020).

Single nucleotide polymorphism (SNP) is a kind of DNA polymorphism in a genome which results from a single nucleotide change in a DNA sequence (Drenkard et al., 2000; Vignal et al., 2002). Because amino acid substitution caused by single nucleotide mutation may change protein function to some extent, single nucleotide changes provide new insights into protein function (Henikoff and Comai, 2003). Specific single nucleotide change can alter protein function, which is closely related with agronomic traits, then was used as an important tool for crops genetic improvement (You et al., 2018; Zhang et al., 2018). Functional markers derived from polymorphic sites within genes causally affect phenotypic variation. Functional markers are superior to random DNA markers such as RFLPs, SSRs and AFLPs owing to complete linkage with trait locus alleles, and are considered to be more accurate and efficient for gene identification and marker-aided selection (Andersen and Lubberstedt, 2003; Varshney et al., 2005; Zhou et al., 2013; Li et al., 2022). Over the past few decades, allele-specific PCR (AS PCR) markers, cleaved amplified polymorphic sequences (CAPS) markers, derived CAPS (dCAPS) markers and loop-mediated isothermal amplification (LAMP) markers were developed in plants based on single nucleotide polymorphisms (Michaels and Amasino, 1998; Drenkard et al., 2000; Zhou et al., 2013; Pan et al., 2014; Guo et al., 2020; Wu et al., 2020; Wang et al., 2022). All these markers are used to detect and select interesting traits by differentiating between homozygous and heterozygous states of plants. However, these markers require fragments separation by electrophoresis and/or digestion with restriction enzyme after PCR amplification, making their application relatively limited due to higher costs and lower throughput.

The development of user-friendly tools and platforms makes the wide-scale use and application of SNP markers possible in breeding programs. The KASP (kompetitive allele-specific PCR) genotyping assay utilizes a unique form of competitive allele-specific PCR combined with a novel, homogeneous, fluorescence-based reporting system for the identification and measurement of genetic variation occurring at the nucleotide level to detect SNPs (He et al., 2014). With the advantages of being low-cost and high throughput for genotyping SNPs, the KASP technology has been extensively used in the fields of human, animal and plant genetics (He et al., 2014; Semagn et al., 2013).

In this study, we aimed to develop the KASP assays for high-throughput genotyping for herbicide resistance. The SNPs were identified on the basis of Sanger sequencing of cloned *BnALS1* and *BnALS3* genes from both the resistant 5N and susceptible cultivars of *B. napus*. Allele-specific assays were developed on the basis of SNPs at positions 1676 and 1667 bp from *BnALS1* and *BnALS3*, respectively. The practical utility of the developed KASP assays was established by validating these in three segregating backcross progeny populations varying for herbicide resistance.

## Materials and methods

### Plant material

Three elite semi-winter *B. napus* cultivars (namely ZY50, ZY51 and ZS72) in Zhejiang province of China, and a double mutant 5N (*als1als1/als3als3*) with herbicide resistance (Guo et al., 2020) were used in this study. The seeds were sown usually in late September or early October and harvested around late May in Yangdu, Haining, Zhejiang Province.

### Development of segregation populations with herbicide-resistant 5N

**Figure 1:**
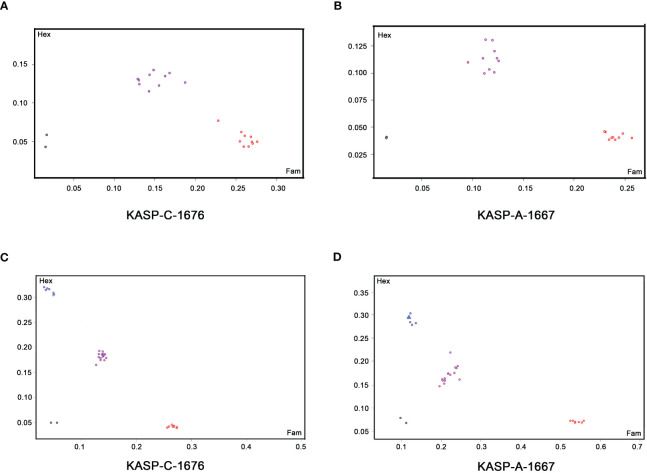
Kompetitive allele-specific PCR (KASP) genotyping using functional marker KASP-C-1676 and KASP-A-1667 in the segregating rapeseed populations for the selection of herbicide-resistant lines. **(A, B)** KASP genotyping of *BnALS1* gene on BC_1_F_1_ lines **(A)** and *BnALS3* gene on BC_1_F_1_ lines **(B)** respectively. The red, purple and black dots represent homozygous alleles (G/G), heterozygous alleles (G/T) and non-template control, respectively. **(C, D)** KASP genotyping of *BnALS1* gene on BC_1_F_2_ lines **(C)** and *BnALS3* gene on BC_1_F_2_ lines **(D)** respectively. The blue, red, purple and black dots represent homozygous alleles (T/T), homozygous alleles (G/G), heterozygous alleles (G/T) and non-template control, respectively.

To obtain herbicide-resistant rapeseed lines with good agronomic and quality traits, three backcross progenies (BC_1_s) were developed from crosses of ZY50/5N//ZY50, ZY51/5N//ZY51 and ZS72/5N//ZS72. The heterozygous lines (*ALS1als1/ALS3als3*) from these three BC_1_F_1_ populations were then screened using newly developed KASP markers (**Table 1**; **Figures 1A, B**) and self-pollinated to produce three distinct BC_1_F_2_ populations for further analysis. The plants were cultivated in the experimental fields located in Yangdu, Haining, Zhejiang province.

**Table 1:** List of primer sequences used for KASP assays.

Gene	Allele	Primer	Sequence (5’-3’)
*BnALS1*	G/T	KASP-C-1676-COM	TGGCGAACCCTGATGCGATTGTTGTGGAT
		KASP-C-1676-HEX	GAAGGTCGGAGTCAACGGATTTAGCTTTGTAGAACCGATCTTCCA
		KASP-C-1676-FAM	GAAGGTGACCAAGTTCATGCTGCTTTGTAGAACCGATCTTCCC
*BnALS3*	G/T	KASP-A-1667-COM	TGGCGAACCCTGATGCGATTGTTGTGGAC
		KASP-A-1667-HEX	GAAGGTCGGAGTCAACGGATTTAGCTTTGTAGAACCGATCTTCCA
		KASP-A-1667-FAM	GAAGGTGACCAAGTTCATGCTGCTTTGTAGAACCGATCTTCCC

### Amplification and Sequence analysis of *BnALS1* and *BnALS3* Genes

Genomic DNA of young rapeseed leaves from each plant was extracted with a modified cetyltriethylammnonium bromide (CTAB) method (Shi et al., 2017). Full-length *BnALS1* (2228 bp) and *BnALS3* (2027 bp) genes were isolated and amplified separately from ZY50, ZY51, ZS72 and 5N using gene-specific primers as described (Guo et al., 2020). The resultant DNA fragments were sequenced by the Sanger dideoxy chain termination method on a capillary electrophoresis system (ABI 3730XL, Applied Biosystems, United States).

Nucleotide and amino acid multiple-sequence alignments were constructed using the CLUSTAL OMEGA program (Madeira et al., 2022) and colored by use of the GeneDoc 3.2 program with the default BLOSUM score. The sequence of the *ALS* gene from *A. thaliana* (GenBank accession no. NM_114714) was used as a reference. The nucleotide and amino acid sequences of *BnALS1* and *BnALS3* from three cultivars and 5N are listed in **Supplementary data sheets 1**, **2**.

### Primer design for *BnALS1* and *BnALS3* genes

*BnALS1* and *BnALS3* sequences from susceptible cultivars (ZY50, ZY51 and ZS72) and the double mutant 5N were amplified and analyzed as mentioned above. Two herbicide-resistant SNPs, G1676T for *BnALS1* and G1667T for *BnALS3*, were used to develop functional markers. The KASP primers were designed according to the standard guidelines. Because *BnALS1* and *BnALS3* sequences are highly identical (97.6%), flanking sequence (including SNPs between *BnALS1* and *BnALS3*) of different alleles at each locus were extracted and used for primer design (**Supplementary Figure 1**). For each gene, the KASP marker consisted of two SNP-specific primers and one common primer. Of these three primers, two G/T alleles were linked to the FAM and HEX fluorescent linker-specific sequence of the LGC KASP reagents at the 5’ end. The primer sequences are shown in **Table 1**.

### Kompetitive allele-specific PCR genotyping

The genotyping assays of the developed KASP markers were performed on a 96-well plate. The KASP assay was performed in a 1.6 μL PCR reaction mix that consisted of 0.8 μL of KASP Master mix (LGC, Biosearch Technologies), approximately equal to 0.05 μL of primer, and 0.8 μL of DNA at a concentration of 10-20 ng/μL. The amplifications were performed using an IntelliQube (LGC, Biosearch Technologies) with the following cycling conditions: 94°C for 15min, 10 touchdown cycles (94°C for 20 s; touchdown at 61°C, dropping to -0.6°C per cycle 60 s) and followed by 26 cycles of amplification (94°C for 20 s, 55°C for 60 s).

### Inheritance analysis

The susceptible rapeseed cultivars (ZY50, ZY51 and ZS72), 5N and the developed distinct BC_1_F_2_ populations were grown in the field, and seedlings at the 4-6 leaf stage were sprayed with tribenuron-methyl (TBM) at 20.25 g.a.i.ha^-1^. Resistance of the parents and their derived BC_1_F_2_ populations was evaluated 20 days after treatment. The response phenotypes were scored as resistant (R) if they showed no herbicide damage or only slight injury, or susceptible (S) if they died. The segregation of each population was assessed using a Chi square test.

### Further herbicide resistance analysis of the homozygous genotypes

Three distinct BC_1_F_2_ populations were derived from the crosses ZY50/5N//ZY50, ZY51/5N//ZY51 and ZS72/5N//ZS72. For each population, the seedlings of BC_1_F_2_ populations was analyzed for the four homozygous genotypes (AABB, AAbb, aaBB and aabb) using the composite KASP markers. These homozygous lines were then self-pollinated to generate BC_1_F_3_ seeds.

These BC_1_F_3_ homozygous lines from the three distinct BC_1_F_2_ populations were sown and grown in plastic pots (diameter, 10cm) containing a 1:1:1 mixture of peat moss, perlite and vermiculite under natural light conditions.

At least twenty BC_1_F_3_ seedlings from each of the four homozygous lines from the three distinct BC_1_F_2_ populations were sprayed with serial concentrations of 20.25, 30.38, 40.50 and 135 g.a.i.ha^-1^TBM at the 4-6 leaf stage. Symptoms were recorded as resistant (R - no herbicide damage or only slight injury), mid-resistant (M - chlorosis or necrosis on some leaves, but no death) or susceptible (S - dead plants) at 20 days after the treatment.

## Results

### Phenotypic symptom of herbicide resistance

**Figure 2:**
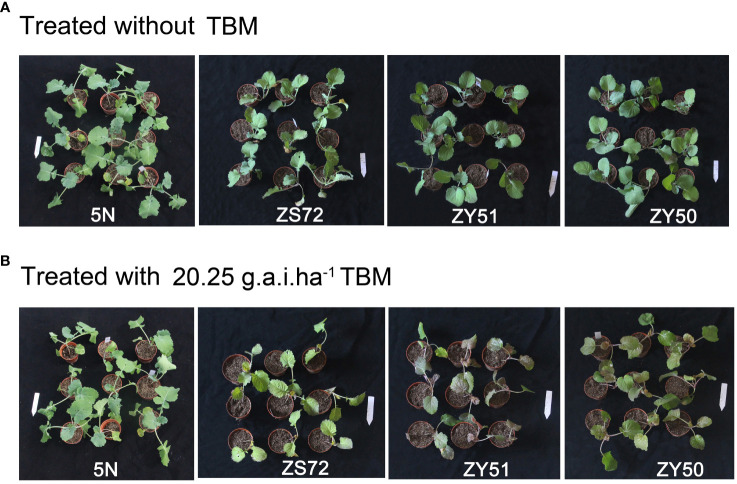
Herbicide resistance phenotypes of three cultivars ZY50, ZY51 and ZS72 and 5N mutant. **(A)** Phenotypes of cultivars ZY50, ZY51 and ZS72 and 5N mutant grown under normal condition. **(B)** Symptoms of cultivars ZY50, ZY51 and ZS72 and 5N mutant 14 days after treatment with 20.25 g.a.i.ha^-1^ TBM.

To observe the resistance to herbicide, the seedlings of ZY50, ZY51, ZS72 and 5N were sprayed with TBM at a concentration of 20.25 g.a.i.ha^-1^. After exposure to TBM for 14 days, ZY50, ZY51 and ZS72 were growth injured with yellow or chlorotic leaves (**Figures 2A, B**). However, 5N, which harbored two resistant alleles, exhibited complete resistance, having no symptoms of chlorosis or necrosis (**Figures 2A, B**). Our results suggested that novel herbicide-resistant materials with good agronomic and quality traits could be developed through the crosses between the elite rapeseed cultivars and 5N.

### Development of kompetitive allele-specific PCR marker for *BnALS1* and *BnALS3* genes

**Figure 3:**
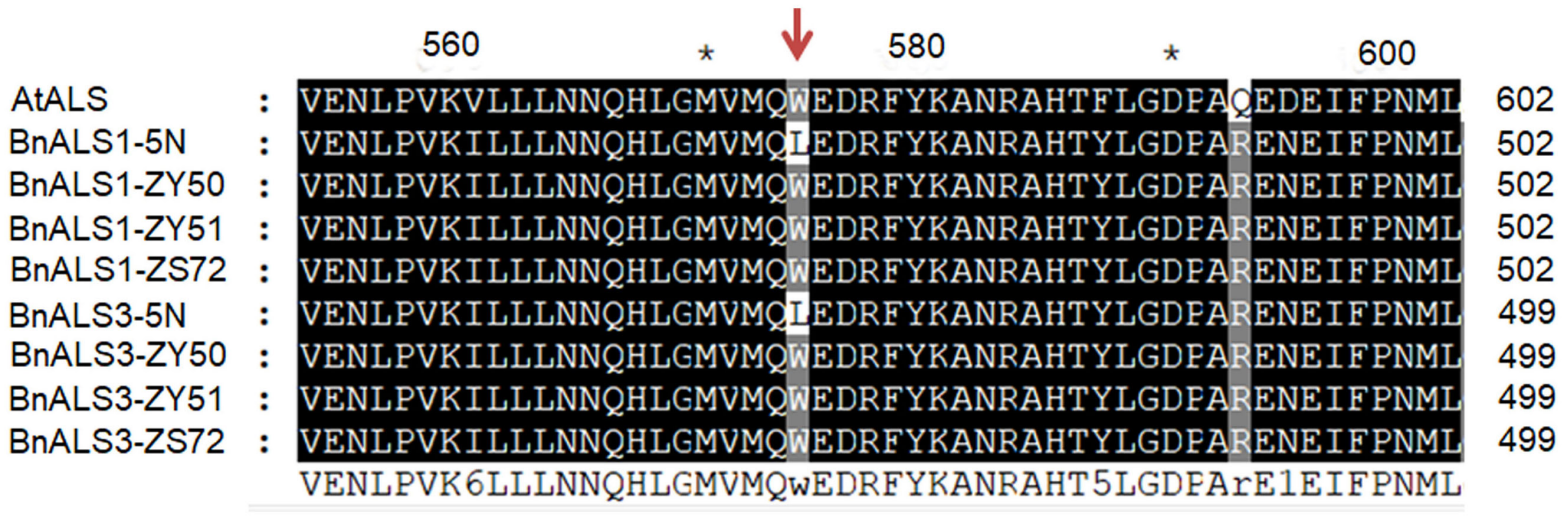
Alignment of partial amino acid sequence of ALS proteins from Arabidopsis, ZY50, ZY51, ZS72 and herbicide-resistant mutant 5N (als1als1/als3als3). AtALS (GenBank accession no. NM_114714), BnALS1-ZY50, BnALS1-ZY51, BnALS1-ZS72, BnALS1-5N, BnALS3-ZY50, BnALS3-ZY51, BnALS3-ZS72 and BnALS3-5N (B. napus L. cv. ZY50, ZY51, ZS72 and 5N). The red arrow represents point mutations occurred in ALS1 and ALS3 of 5N. The shading of the alignment presents as follows: identical residues in black and different residues in dark gray. “*”indicates positions which have a single, fully conserved residue.

To detect single nucleotide polymorphisms (SNPs), *BnALS1* and *BnALS3* genes were separately cloned by PCR amplification from 5N and three cultivars, ZY50, ZY51 and ZS72 (**Supplementary data sheet 1**). Compared with 5N, these three cultivars have a common SNP at position G/T (1676) in *BnALS1* and a common SNP at position G/T (1667) in *BnALS3* (**Supplementary Figure 1**). A comparison of the amino acid sequences of susceptible cultivars/resistant 5N showed changes at W/L (474 in BnALS1; 471 in BnALS3) as compared to the changes at the two positions for the nucleotide sequence of *BnALS1* and *BnALS3* (**Figure 3**). A previous study proved that the substitutions of W/L in BnALS1 and BnALS3 could endow high herbicide resistance (Guo et al., 2020). Therefore, G1676T and G1667T were selected as the genotyping targets for *BnALS1* and *BnALS3* respectively. The KASP markers were designed for a SNP at position 1676 in *BnALS1* and for a SNP at position 1667 in *BnALS3* (**Supplementary Figure 1**). Both the marker KASP-C-1676 (specific to G1676T in *BnALS1*) and the marker KASP-A-1667 (specific to G1667 in *BnALS3*) could clearly distinguish type alleles GG, GT and TT among cultivars, cultivars/5N and 5N (**Supplementary Figure 2**). These two markers were also validated on BC_1_F_1_ populations, and formed separate clusters for heterozygous (GT) and homozygous (GG) alleles (**Figures 1A, B**).

### Validation of kompetitive allele-specific PCR assays on BC_1_F_2_ populations

To confirm the KASP assay on herbicide resistance, three distinct BC_1_F_2_ populations were genotyped using KASP-C-1676 and KASP-A-1667. DNA was extracted from the first true leaves of seedlings before the herbicide treatment. Seedlings at the 4-6 leaf stage were sprayed with TBM at a concentration of 20.25 g.a.i.ha^-1^. Phenotypic symptoms were observed at 20 days after the treatment. The KASP assays were used for specific amplification of *BnALS1* and *BnALS3*. The frequency of the KASP alleles showed equivalence with the segregation expected for the BC_1_F_2_ populations (**Figures 1C, D**).

The combination of the KASP markers resulted in nine genotypes as shown by analysis of the seedlings in the BC_1_F_2_ populations (**Table 2**). These were AABB, AABb, AAbb, AaBB, AaBb, Aabb, aaBB, aaBb and aabb, and the ratio of isolation of these genotypes is 1:2:1:2:4:2:1:2:1 in the three distinct BC_1_F_2_ populations. In the three BC_1_F_2_ populations developed from crosses ZY50/5N//ZY50, ZY51/5N//ZY51 and ZS72/5N//ZS72, five, five and six homozygous plants with genotype AABB exhibited sensitivity to TBM treatment at 20 days after the treatment (**Table 2**). However, plants with other genotypes showed resistance to 20.25 g.a.i.ha^-1^ TBM treatment (**Table 2**). The ratio of susceptible lines to resistant lines is 1:15 in the three distinct BC_1_F_2_ populations (**Table 2**).

**Table 2:** Validation of the KASP assays for herbicide resistance in distinct BC_1_F_2_ populations of *B. napus*.

Genotype	BC_1_F_2_ (No of samples)			Phenotype
	ZY50/5N//ZY50	ZY51/5N//ZY51	ZS72/5N//ZS72	
AABB	5	5	6	S
AABb	24	24	20	R
AAbb	9	10	7	R
AaBB	16	16	20	R
AaBb	54	34	44	R
Aabb	22	22	27	R
aaBB	8	12	12	R
aaBb	18	17	24	R
aabb	4	6	9	R
Total	160	146	169	–
*P* value (1:2:1:2:4:2:1:2:1)	0.0958	0.5322	0.6678	–
*χ*2	13.5	7.0411	5.8166	–
–	0.0875	0.0875	0.0770	*P* value (1:15)
–	2.9192	2.9192	3.1277	*χ*2

A/a represents herbicide susceptible/resistant allele for BnALS1 gene; B/b represents herbicide susceptible/resistant allele for BnALS3 gene. The seedlings were treated with 20.25 g.a.i.ha^-1^ TBM at the 4-6 leaf stage, and phenotypes of the seedlings were observed 20 days after the treatment. R represents resistant to TBM; S represents susceptible to TBM.

### Further validation of genotype effect on herbicide resistance

To further validate the effect of genotype on herbicide resistance, we chose the BC_1_F_3_ plants with genotypes AABB, AAbb, aaBB and aabb developed from three distinct BC_1_F_2_ populations for resistance analysis. Plants with genotype AABB displayed serious damage with yellow leaves and eventual death within 20 days after treatment at all concentrations of TBM (**Table 3**). Plants with genotype AAbb exhibited resistance to 20.25-40.50 g.a.i.ha^-1^ TBM (**Table 3**). Plants with genotype aaBB showed resistance to 20.25-30.38 g.a.i.ha^-1^ TBM and mid-resistance to 40.50 g.a.i.ha^-1^ TBM (**Table 3**). However, at higher concentration of 135 g.a.i.ha^-1^ TBM, Plants with genotype AAbb and aaBB showed chlorotic stunting, destroyed apex and eventual death (**Table 3**). By contrast to plants with genotype AAbb and aaBB, plants with genotype aabb exhibited complete resistance, having no chlorosis or necrosis, even to the higher concentration of 135 g.a.i.ha^-1^ TBM (**Table 3**).

**Table 3:** The effects of four homozygous genotypes on herbicide resistance.

Genotype	TBM (g.a.i.ha^-1^)			
	20.25	30.38	40.50	135
AABB	S	S	S	S
AAbb	R	R	R	S
aaBB	R	R	M	S
aabb	R	R	R	R

A/a represents herbicide susceptible/resistant allele for BnALS1 gene; B/b represents herbicide susceptible/resistant allele for BnALS3 gene. R represents resistant to TBM;.

M represents mid-resistant to TBM; S represents susceptible to TBM.

## Discussion

Successful weed management helps to improve crop yield in modern agricultural production systems. Resistant cultivars are the most effective and environmentally responsible strategy for protecting crops from weeds. Thus, developing new cultivars with high resistance to herbicides is now a major breeding objective in rapeseed. Acetolactate synthase encoded by *ALS* gene is responsible for biosynthesis of the branched chain amino acids, including valine, leucine, and isoleucine (Haughn and Somerville, 1990). The mutation of *ALS* gene may result in amino acid substitutions of ALS and inhibit the binding of the ALS enzyme with herbicides, which endows the plants with resistance to herbicide (Duggleby et al., 2008; Murphy and Tranel, 2019; Guo et al., 2020; Wu et al., 2020). Functional markers, such as AS-PCR (Hu et al., 2015) and CAPS (Li et al., 2015; Hu et al., 2017; Guo et al., 2020; Huang et al., 2020), have been developed to discriminate the allelic variation for the *ALS* genes. However, all these are gel based markers, and have relatively limited potential for high-throughput application. Thus, the development of a high-throughput and relatively cost-efficient marker system is important and necessary for improving breeding strategies.

As a key enzyme for the biosynthesis of branched chain amino acids, improper mutation of *ALS* can destroy its function and result in plant death. However, *ALS* harboring point mutations could confer sufficient tolerance to some kinds of herbicides with little damage to plant growth (Yu et al., 2010; Zhao et al., 2020; Cheng et al., 2021). We independently cloned and sequenced *BnALS1* and *BnALS3* from three cultivars ZY50, ZY51 and ZS72, and from the 5N mutant. DNA sequence alignment showed that 5N contains a single-nucleotide mutation (G1676T) in *BnALS1* and a single- nucleotide mutation (G1667T) in *BnALS3* based on sequence comparison with the three herbicide-susceptible cultivars; ZY50, ZY51 and ZS72 (**Supplementary Figure 1**), resulting in amino acid alterations, W474L (W574L, numbered according to ALS sequence in Arabidopsis) in BnALS1 and W471L (W574L) in BnALS3 (**Figure 2**). The W574L substitution has been reported to confer resistance to ALS inhibitors in rapeseed, sunflower and cocklebur (Bernasconi et al., 1995; Hattori et al., 1995; Sala et al., 2012; Hu et al., 2017; Guo et al., 2020). Mutation at P197 also conferred good tolerance to sulfonylureas in Arabidopsis, rapeseed and wheat (Li et al., 2015; Chen et al., 2017; Zhang et al., 2019; Huang et al., 2020; Wu et al., 2020; Cheng et al., 2021; Guo et al., 2022). In addition, mutations at the sites of Ala122, Ala205 and Ser653 of ALS have been reported to confer resistance to ALS inhibitors (Tan et al., 2005; Murphy and Tranel, 2019). These SNPs in *ALS* genes can be used for marker-assisted breeding.

5N is an important herbicide-resistant material with simultaneous mutations in *BnALS1* and *BnALS3* genes (Guo et al., 2020). We planned to design KASP markers for *BnALS1* and *BnALS3* genes in the 5N double mutant. Considering the highly similar (97.6%) sequence of *BnALS1* and *BnALS3*, it is difficult to develop high throughput markers capable of discriminating homozyous and heterozygous lines in these segregating populations. In this study, two KASP functional markers, KASP-C-1676 and KASP-A-1667, were successfully developed based on the specific characteristics of *BnALS1* and *BnALS3* genes from the cultivars and 5N (**Table 1**). These KASP markers will faciliate the use of 5N mutant for herbicide resistant rapeseed breeding.

The two KASP markers can clearly distinguish the genotypes of parents and hybrids (**Supplementary Figure 2**). Genotyping results performed by the two markers are highly consistent with the results of phenotypic evaluation (**Figure 1**; **Table 2**). Furthermore, these two KASP markers can distinguish the homozygous/heterozygous lines in three distinct segregated populations (BC_1_F_2_) developed from ZY50, ZY51, ZS72 and 5N (**Table 2**), which proved the high effectiveness of the KASP markers for genotyping under different genetic backgrounds. All these results suggested that the developed KASP markers are stable and effective to differentiate homozygous/heterozygous state of alleles in distinct populations and can be used for marker-assisted selection in rapeseed breeding projects.

In plants, synergistic effect is an important genetic phenomenon exhibited in the processes of hormone interaction, flower development and signal transduction (Poduska et al., 2003; Replogle et al., 2013; Yang et al., 2017). Synergistic effects have also been shown for herbicide resistance in crops. In *B. napus*, 5N (BnALS1-2R, W574L; BnALS3R, W574L) and DS3 (BnALS1-3R, P197L; BnALS3R, W574L) showed stronger herbicide resistance than mutants with single-point mutations (Guo et al., 2020 and Guo et al., 2022). In soybean, *Als1* (P197S) and *Als2* (W574L) exhibited synergistic resistant effects to ALS herbicides, and the combination of *Als1 and Als2* conferred stronger tolerance to SU (Walter et al., 2014). In this study, four homozygous genotypes were characterized and selected using the developed KASP markers. We analyze the effects of four genotypes on herbicide resistance. Our results showed that the lines containing two mutated alleles exhibited relatively stronger TBM resistance compared with those lines with a single mutated allele (**Table 3**), which is consistent with the findings reported previously (Guo et al., 2020). Altogether, these results suggested that the developed KASP markers are valuable functional markers and could be used for the high throughput selection of superior herbicide resistant materials by providing precise genotypic information, which will expedite the process of breeding herbicide-resistant rapeseed.

## Conclusion

In this study, two KASP markers for *BnALS1* and *BnALS3*, KASP-C-1676 and KASP-A-1667, were successfully developed on the basis of SNPs in the *ALS* genes. These assays are highly gene specific and can effectively distinguish target genotype states. The developed KASP assays are high throughput and cost effective as compared to gel-based markers and can be used for marker-assisted selection of herbicide resistance.

## Data availability statement

The datasets presented in this study can be found in online repositories. The names of the repository/repositories and accession number(s) can be found in the article/**Supplementary Material**.

## Author contributions

JS conceived and coordinated the study. TW cloned and aligned the genes. TZ, YF, and SL conducted the KASP assays. XN, HY, YZ, and JH performed the field experiments and phenotypic data collection. JS wrote the manuscript and JZ revised it. All authors contributed to the article and approved the submitted version.
