# Supplementary material for: Development and validation of functional kompetitive allele-specific PCR markers for herbicide resistance in Brassica napus
Source: Front Plant Sci. 2023 Nov 23;14:1213476. doi: 10.3389/fpls.2023.1213476 (PMC10701909; doi:10.3389/fpls.2023.1213476)
Supplement: Supplementary file 1 [file DataSheet_1.docx]

>***AtALS***

ATGGCGGCGGCAACAACAACAACAACAACATCTTCTTCGATCTCCTTCTCCACCAAACCATCTCCTTCCTCCTCCAAATCACCATTACCAATCTCCAGATTCTCCCTCCCATTCTCCCTAAACCCCAACAAATCATCCTCCTCCTCCCGCCGCCGCGGTATCAAATCCAGCTCTCCCTCCTCCATCTCCGCCGTGCTCAACACAACCACCAATGTCACAACCACTCCCTCTCCAACCAAACCTACCAAACCCGAAACATTCATCTCCCGATTCGCTCCAGATCAACCCCGCAAAGGCGCTGATATCCTCGTCGAAGCTTTAGAACGTCAAGGCGTAGAAACCGTATTCGCTTACCCTGGAGGTGCATCAATGGAGATTCACCAAGCCTTAACCCGCTCTTCCTCAATCCGTAACGTCCTTCCTCGTCACGAACAAGGAGGTGTATTCGCAGCAGAAGGATACGCTCGATCCTCAGGTAAACCAGGTATCTGTATAGCCACTTCAGGTCCCGGAGCTACAAATCTCGTTAGCGGATTAGCCGATGCGTTGTTAGATAGTGTTCCTCTTGTAGCAATCACAGGACAAGTCCCTCGTCGTATGATTGGTACAGATGCGTTTCAAGAGACTCCGATTGTTGAGGTAACGCGTTCGATTACGAAGCATAACTATCTTGTGATGGATGTTGAAGATATCCCTAGGATTATTGAGGAAGCTTTCTTTTTAGCTACTTCTGGTAGACCTGGACCTGTTTTGGTTGATGTTCCTAAAGATATTCAACAACAGCTTGCGATTCCTAATTGGGAACAGGCTATGAGATTACCTGGTTATATGTCTAGGATGCCTAAACCTCCGGAAGATTCTCATTTGGAGCAGATTGTTAGGTTGATTTCTGAGTCTAAGAAGCCTGTGTTGTATGTTGGTGGTGGTTGTTTGAATTCTAGCGATGAATTGGGTAGGTTTGTTGAGCTTACGGGGATCCCTGTTGCGAGTACGTTGATGGGGCTGGGATCTTATCCTTGTGATGATGAGTTGTCGTTACATATGCTTGGAATGCATGGGACTGTGTATGCAAATTACGCTGTGGAGCATAGTGATTTGTTGTTGGCGTTTGGGGTAAGGTTTGATGATCGTGTCACGGGTAAGCTTGAGGCTTTTGCTAGTAGGGCTAAGATTGTTCATATTGATATTGACTCGGCTGAGATTGGGAAGAATAAGACTCCTCATGTGTCTGTGTGTGGTGATGTTAAGCTGGCTTTGCAAGGGATGAATAAGGTTCTTGAGAACCGAGCGGAGGAGCTTAAGCTTGATTTTGGAGTTTGGAGGAATGAGTTGAACGTACAGAAACAGAAGTTTCCGTTGAGCTTTAAGACGTTTGGGGAAGCTATTCCTCCACAGTATGCGATTAAGGTCCTTGATGAGTTGACTGATGGAAAAGCCATAATAAGTACTGGTGTCGGGCAACATCAAATGTGGGCGGCGCAGTTCTACAATTACAAGAAACCAAGGCAGTGGCTATCATCAGGAGGCCTTGGAGCTATGGGATTTGGACTTCCTGCTGCGATTGGAGCGTCTGTTGCTAACCCTGATGCGATAGTTGTGGATATTGACGGAGATGGAAGCTTTATAATGAATGTGCAAGAGCTAGCCACTATTCGTGTAGAGAATCTTCCAGTGAAGGTACTTTTATTAAACAACCAGCATCTTGGCATGGTTATGCAATGGGAAGATCGGTTCTACAAAGCTAACCGAGCTCACACATTTCTCGGGGATCCGGCTCAGGAGGACGAGATATTCCCGAACATGTTGCTGTTTGCAGCAGCTTGCGGGATTCCAGCGGCGAGGGTGACAAAGAAAGCAGATCTCCGAGAAGCTATTCAGACAATGCTGGATACACCAGGACCTTACCTGTTGGATGTGATTTGTCCGCACCAAGAACATGTGTTGCCGATGATCCCGAGTGGTGGCACTTTCAACGATGTCATAACGGAAGGAGATGGCCGGATTAAATACTGA

>***BnALS1-Zheyou50***

ATGGCGGCGGCAACATCGTCTTCTCCGATCTCCTTAACCGCTAAACCTTCTTCCAAATCCCCTCTACCCATTTCCAGATTCTCCCTTCCCTTCTCCTTAACCCCACAGAAAGACTCCTCCCGTCTCCACCGTCCTCTCGCCATCTCCGCCGTTCTCAACTCACCCGTCAATGTCGCACCTCCTTCCCCTGAAAAAACCGACAAGAACAAGACTTTCGTCTCCCGCTACGCTCCCGACGAGCCCCGCAAGGGTGCTGATATCCTCGTCGAAGCCCTCGAGCGTCAAGGCGTCGAAACCGTCTTTGCTTATCCCGGAGGTGCTTCCATGGAGATCCACCAAGCCTTGACTCGCTCCTCCACCATCCGTAACGTCCTTCCCCGTCACGAACAAGGAGGAGTCTTCGCCGCCGAGGGTTACGCTCGTTCCTCCGGCAAACCGGGAATCTGCATAGCCACTTCGGGTCCCGGAGCTACCAACCTCGTCAGCGGGTTAGCAGACGCGATGCTTGACAGTGTTCCTCTTGTCGCCATTACAGGACAGGTCCCTCGCCGGATGATCGGTACTGACGCCTTCCAAGAGACACCAATCGTTGAGGTAACGAGGTCTATTACGAAACATAACTATTTGGTGATGGATGTTGATGACATACCTAGGATCGTTCAAGAAGCTTTCTTTCTAGCTACTTCCGGTAGACCCGGACCGGTTTTGGTTGATGTTCCTAAGGATATTCAGCAGCAGCTTGCGATTCCTAACTGGGATCAACCTATGCGCTTACCTGGCTACATGTCTAGGTTGCCTCAGCCTCCGGAAGTTTCTCAGTTAGGTCAGATCGTTAGGTTGATCTCGGAGTCTAAGAGGCCTGTTTTGTACGTTGGTGGTGGAAGCTTGAACTCGAGTGAAGAACTGGGGAGATTTGTCGAGCTTACTGGGATCCCCGTTGCGAGTACTTTGATGGGGCTTGGCTCTTATCCTTGTAACGATGAGTTGTCCCTGCAGATGCTTGGCATGCACGGGACTGTGTATGCTAACTACGCTGTGGAGCATAGTGATTTGTTGCTGGCGTTTGGTGTTAGGTTTGATGACCGTGTCACGGGAAAGCTCGAGGCTTTCGCTAGCAGGGCTAAAATTGTGCACATAGACATTGATTCTGCTGAGATTGGGAAGAATAAGACACCTCACGTGTCTGTGTGTGGTGATGTAAAGCTGGCTTTGCAAGGGATGAACAAGGTTCTTGAGAACCGGGCGGAGGAGCTCAAGCTTGATTTCGGTGTTTGGAGGAGTGAGTTGAGCGAGCAGAAACAGAAGTTCCCTTTGAGCTTCAAAACGTTTGGAGAAGCCATTCCTCCGCAGTACGCGATTCAGATCCTCGACGAGCTAACCGAAGGGAAGGCAATTATCAGTACTGGTGTTGGACAGCATCAGATGTGGGCGGCGCAGTTTTACAAGTACAGGAAGCCGAGACAGTGGCTGTCGTCATCAGGCCTCGGAGCTATGGGTTTTGGACTTCCTGCTGCGATTGGAGCGTCTGTGGCGAACCCTGATGCGATTGTTGTGGATATTGACGGTGATGGAAGCTTCATAATGAACGTTCAAGAGCTGGCCACAATCCGTGTAGAGAATCTTCCTGTGAAGATACTCTTGTTAAACAACCAGCATCTTGGGATGGTCATGCAATGGGAAGATCGGTTCTACAAAGCTAACAGAGCTCACACTTATCTCGGGGACCCGGCAAGGGAGAACGAGATCTTCCCTAACATGCTGCAGTTTGCAGGAGCTTGCGGGATTCCAGCTGCGAGAGTGACGAAGAAAGAAGAACTCCGAGAAGCTATTCAGACAATGCTGGATACACCAGGACCATACCTGTTGGATGTGATATGTCCGCACCAAGAACATGTGTTACCGATGATCCCAAGTGGTGGCACTTTCAAAGATGTAATAACAGaaggggatggtcgcactaagtactga

>***BnALS1-Zheyou51***

ATGGCGGCGGCAACATCGTCTTCTCCGATCTCCTTAACCGCTAAACCTTCTTCCAAATCCCCTCTACCCATTTCCAGATTCTCCCTTCCCTTCTCCTTAACCCCACAGAAAGACTCCTCCCGTCTCCACCGTCCTCTCGCCATCTCCGCCGTTCTCAACTCACCCGTCAATGTCGCACCTCCTTCCCCTGAAAAAACCGACAAGAACAAGACTTTCGTCTCCCGCTACGCTCCCGACGAGCCCCGCAAGGGTGCTGATATCCTCGTCGAAGCCCTCGAGCGTCAAGGCGTCGAAACCGTCTTTGCTTATCCCGGAGGTGCTTCCATGGAGATCCACCAAGCCTTGACTCGCTCCTCCACCATCCGTAACGTCCTTCCCCGTCACGAACAAGGAGGAGTCTTCGCCGCCGAGGGTTACGCTCGTTCCTCCGGCAAACCGGGAATCTGCATAGCCACTTCGGGTCCCGGAGCTACCAACCTCGTCAGCGGGTTAGCAGACGCGATGCTTGACAGTGTTCCTCTTGTCGCCATTACAGGACAGGTCCCTCGCCGGATGATCGGTACTGACGCCTTCCAAGAGACACCAATCGTTGAGGTAACGAGGTCTATTACGAAACATAACTATTTGGTGATGGATGTTGATGACATACCTAGGATCGTTCAAGAAGCTTTCTTTCTAGCTACTTCCGGTAGACCCGGACCGGTTTTGGTTGATGTTCCTAAGGATATTCAGCAGCAGCTTGCGATTCCTAACTGGGATCAACCTATGCGCTTACCTGGCTACATGTCTAGGTTGCCTCAGCCTCCGGAAGTTTCTCAGTTAGGTCAGATCGTTAGGTTGATCTCGGAGTCTAAGAGGCCTGTTTTGTACGTTGGTGGTGGAAGCTTGAACTCGAGTGAAGAACTGGGGAGATTTGTCGAGCTTACTGGGATCCCCGTTGCGAGTACTTTGATGGGGCTTGGCTCTTATCCTTGTAACGATGAGTTGTCCCTGCAGATGCTTGGCATGCACGGGACTGTGTATGCTAACTACGCTGTGGAGCATAGTGATTTGTTGCTGGCGTTTGGTGTTAGGTTTGATGACCGTGTCACGGGAAAGCTCGAGGCTTTCGCTAGCAGGGCTAAAATTGTGCACATAGACATTGATTCTGCTGAGATTGGGAAGAATAAGACACCTCACGTGTCTGTGTGTGGTGATGTAAAGCTGGCTTTGCAAGGGATGAACAAGGTTCTTGAGAACCGGGCGGAGGAGCTCAAGCTTGATTTCGGTGTTTGGAGGAGTGAGTTGAGCGAGCAGAAACAGAAGTTCCCTTTGAGCTTCAAAACGTTTGGAGAAGCCATTCCTCCGCAGTACGCGATTCAGATCCTCGACGAGCTAACCGAAGGGAAGGCAATTATCAGTACTGGTGTTGGACAGCATCAGATGTGGGCGGCGCAGTTTTACAAGTACAGGAAGCCGAGACAGTGGCTGTCGTCATCAGGCCTCGGAGCTATGGGTTTTGGACTTCCTGCTGCGATTGGAGCGTCTGTGGCGAACCCTGATGCGATTGTTGTGGATATTGACGGTGATGGAAGCTTCATAATGAACGTTCAAGAGCTGGCCACAATCCGTGTAGAGAATCTTCCTGTGAAGATACTCTTGTTAAACAACCAGCATCTTGGGATGGTCATGCAATGGGAAGATCGGTTCTACAAAGCTAACAGAGCTCACACTTATCTCGGGGACCCGGCAAGGGAGAACGAGATCTTCCCTAACATGCTGCAGTTTGCAGGAGCTTGCGGGATTCCAGCTGCGAGAGTGACGAAGAAAGAAGAACTCCGAGAAGCTATTCAGACAATGCTGGATACACCAGGACCATACCTGTTGGATGTGATATGTCCGCACCAAGAACATGTGTTACCGATGATCCCAAGTGGTGGCACTTTCAAAGATGTAATAACAGAAGGGGATGGTCGCACTAAGTACTGA

>***BnALS1-Zheshuang72***

ATGGCGGCGGCAACATCGTCTTCTCCGATCTCCTTAACCGCTAAACCTTCTTCCAAATCCCCTCTACCCATTTCCAGATTCTCCCTTCCCTTCTCCTTAACCCCACAGAAAGACTCCTCCCGTCTCCACCGTCCTCTCGCCATCTCCGCCGTTCTCAACTCACCCGTCAATGTCGCACCTCCTTCCCCTGAAAAAATCGACAAGAACAAGACTTTCGTCTCCCGCTACGCTCCCGACGAGCCCCGCAAGGGTGCTGATATCCTCGTCGAAGCCCTCGAGCGTCAAGGCGTCGAAACCGTCTTTGCTTATCCCGGAGGTGCTTCCATGGAGATCCACCAAGCCTTGACTCGCTCCTCCACCATCCGTAACGTCCTTCCCCGTCACGAACAAGGAGGAGTCTTCGCCGCCGAGGGTTACGCTCGTTCCTCCGGCAAACCGGGAATCTGCATAGCCACTTCGGGTCCCGGAGCTACCAACCTCGTCAGCGGGTTAGCAGACGCGATGCTTGACAGTGTTCCTCTTGTCGCCATTACAGGACAGGTCCCTCGCCGGATGATCGGTACTGACGCCTTCCAAGAGACACCAATCGTTGAGGTAACGAGGTCTATTACGAAACATAACTATTTGGTGATGGATGTTGATGACATACCTAGGATCGTTCAAGAAGCTTTCTTTCTAGCTACTTCCGGTAGACCCGGACCGGTTTTGGTTGATGTTCCTAAGGATATTCAGCAGCAGCTTGCGATTCCTAACTGGGATCAACCTATGCGCTTACCTGGCTACATGTCTAGGTTGCCTCAGCCTCCGGAAGTTTCTCAGTTAGGTCAGATCGTTAGGTTGATCTCGGAGTCTAAGAGGCCTGTTTTGTACGTTGGTGGTGGAAGCTTGAACTCGAGTGAAGAACTGGGGAGATTTGTCGAGCTTACTGGGATCCCCGTTGCGAGTACTTTGATGGGGCTTGGCTCTTATCCTTGTAACGATGAGTTGTCCCTGCAGATGCTTGGCATGCACGGGACTGTGTATGCTAACTACGCTGTGGAGCATAGTGATTTGTTGCTGGCGTTTGGTGTTAGGTTTGATGACCGTGTCACGGGAAAGCTCGAGGCTTTCGCTAGCAGGGCTAAAATTGTGCACATAGACATTGATTCTGCTGAGATTGGGAAGAATAAGACACCTCACGTGTCTGTGTGTGGTGATGTAAAGCTGGCTTTGCAAGGGATGAACAAGGTTCTTGAGAACCGAGCGGAGGAGCTCAAGCTTGATTTCGGTGTTTGGAGGAGTGAGTTGAGCGAGCAGAAACAGAAGTTCCCTTTGAGCTTCAAAACGTTTGGAGAAGCCATTCCTCCGCAGTACGCGATTCAGATCCTCGACGAGCTAACCGAAGGGAAGGCAATTATCAGTACTGGTGTTGGACAGCATCAGATGTGGGCGGCGCAGTTTTACAAGTACAGGAAGCCGAGACAGTGGCTGTCGTCATCAGGCCTCGGAGCTATGGGTTTTGGACTTCCTGCTGCGATTGGAGCGTCTGTGGCGAACCCTGATGCGATTGTTGTGGATATTGACGGTGATGGAAGCTTCATAATGAACGTTCAAGAGCTGGCCACAATCCGTGTAGAGAATCTTCCTGTGAAGATACTCTTGTTAAACAACCAGCATCTTGGGATGGTCATGCAATGGGAAGATCGGTTCTACAAAGCTAACAGAGCTCACACTTATCTCGGGGACCCGGCAAGGGAGAACGAGATCTTCCCTAACATGCTGCAGTTTGCAGGAGCTTGCGGGATTCCAGCTGCGAGAGTGACGAAGAAAGAAGAACTCCGAGAAGCTATTCAGACAATGCTGGATACACCAGGACCATACCTGTTGGATGTGATCTGTCCGCACCAAGAACATGTGTTACCGATGATCCCAAGTGGTGGCACTTTCAAAGATGTAATAACAGAAGGGGATGGTCGCACTAAGTACTGA

>***BnALS1-5N***

ATGGCGGCGGCAACATCGTCTTCTCCGATCTCCTTAACCGCTAAACCTTCTTCCAAATCCCCTCTACCCATTTCCAGATTCTCCCTTCCCTTCTCCTTAACCCCACAGAAAGACTCCTCCCGTCTCCACCGTCCTCTCGCCATCTCCGCCGTTCTCAACTCACCCGTCAATGTCGCACCTCCTTCCCCTGAAAAAACCGACAAGAACAAGACTTTCGTCTCCCGCTACGCTCCCGACGAGCCCCGCAAGGGTGCTGATATCCTCGTCGAAGCCCTCGAGCGTCAAGGCGTCGAAACCGTCTTTGCTTATCCCGGAGGTGCTTCCATGGAGATCCACCAAGCCTTGACTCGCTCCTCCACCATCCGTAACGTCCTTCCCCGTCACGAACAAGGAGGAGTCTTCGCCGCCGAGGGTTACGCTCGTTCCTCCGGCAAACCGGGAATCTGCATAGCCACTTCGGGTCCCGGAGCTACCAACCTCGTCAGCGGGTTAGCAGACGCGATGCTTGACAGTGTTCCTCTTGTCGCCATTACAGGACAGGTCCCTCGCCGGATGATCGGTACTGACGCCTTCCAAGAGACACCAATCGTTGAGGTAACGAGGTCTATTACGAAACATAACTATTTGGTGATGGATGTTGATGACATACCTAGGATCGTTCAAGAAGCTTTCTTTCTAGCTACTTCCGGTAGACCCGGACCGGTTTTGGTTGATGTTCCTAAGGATATTCAGCAGCAGCTTGCGATTCCTAACTGGGATCAACCTATGCGCTTACCTGGCTACATGTCTAGGTTGCCTCAGCCTCCGGAAGTTTCTCAGTTAGGTCAGATCGTTAGGTTGATCTCGGAGTCTAAGAGGCCTGTTTTGTACGTTGGTGGTGGAAGCTTGAACTCGAGTGAAGAACTGGGGAGATTTGTCGAGCTTACTGGGATCCCCGTTGCGAGTACTTTGATGGGGCTTGGCTCTTATCCTTGTAACGATGAGTTGTCCCTGCAGATGCTTGGCATGCACGGGACTGTGTATGCTAACTACGCTGTGGAGCATAGTGATTTGTTGCTGGCGTTTGGTGTTAGGTTTGATGACCGTGTCACGGGAAAGCTCGAGGCTTTCGCTAGCAGGGCTAAAATTGTGCACATAGACATTGATTCTGCTGAGATTGGGAAGAATAAGACACCTCACGTGTCTGTGTGTGGTGATGTAAAGCTGGCTTTGCAAGGGATGAACAAGGTTCTTGAGAACCGGGCGGAGGAGCTCAAGCTTGATTTCGGTGTTTGGAGGAGTGAGTTGAGCGAGCAGAAACAGAAGTTCCCTTTGAGCTTCAAAACGTTTGGAGAAGCCATTCCTCCGCAGTACGCGATTCAGATCCTCGACGAGCTAACCGAAGGGAAGGCAATTATCAGTACTGGTGTTGGACAGCATCAGATGTGGGCGGCGCAGTTTTACAAGTACAGGAAGCCGAGACAGTGGCTGTCGTCATCAGGCCTCGGAGCTATGGGTTTTGGACTTCCTGCTGCGATTGGAGCGTCTGTGGCGAACCCTGATGCGATTGTTGTGGATATTGACGGTGATGGAAGCTTCATAATGAACGTTCAAGAGCTGGCCACAATCCGTGTAGAGAATCTTCCTGTGAAGATACTCTTGTTAAACAACCAGCATCTTGGGATGGTCATGCAATTGGAAGATCGGTTCTACAAAGCTAACAGAGCTCACACTTATCTCGGGGACCCGGCAAGGGAGAACGAGATCTTCCCTAACATGCTGCAGTTTGCAGGAGCTTGCGGGATTCCAGCTGCGAGAGTGACGAAGAAAGAAGAACTCCGAGAAGCTATTCAGACAATGCTGGATACACCAGGACCATACCTGTTGGATGTGATATGTCCGCACCAAGAACATGTGTTACCGATGATCCCAAGTGGTGGCACTTTCAAAGATGTAATAACAGAAGGGGATGGTCGCACTAAGTACTGA

>***BnALS3-Zheyou50***

atggcggcggcaacatcgtcttctccgatctccttaaccgctaaaccttcttccaaatcccctctacccatttccagattctcccttcccttctccttaaccccacagaaaccctcctcccgtctccaccgtcctctcgccatctccgccgttctcaactcacccgtcaatgtcgcacctgaaaaaaccgacaagatcaagactttcatctcccgctacgctcccgacgagccccgcaagggtgctgatatcctcgtggaagccctcgagcgtcaaggcgtcgaaaccgtcttcgcttatcccggaggtgcttccatggagatccaccaagccttgactcgctcctccaccatccgtaacgtcctTccccgtcacgaacaaggaggagtcttcgccgccgagggttacgctcgttcctccggcaaaccgggaatctgcatagccacttcgggtcccggagctaccaacctcgtcagcgggttagcAgacgcgatgcttgacagtgttcctctcgtcgccatcacaggacaggtccctcgccggatgatcggtactgacgcgttccaagagacgccaatcgttgaggtaacgaggtctattacgaaacataactatctggtgatggatgttgatgacatacctaggatcgttcaagaagctttctttctagctacttccggtagacccggaccggttttggttgatgttcctaaggatattcagcagcagcttgcgattcctaactgggatcaacctatgcgcttgcctggctacatgtctaggctgcctcagccaccggaagtttctcagttaggtcagatcgttaggttgatctcggagtctaagaggcctgttttgtacgttggtggtggaagcttgaactcgagtgaagaactggggagatttgtcgagcttactgggatccctgttgcgagtacgttgatggggcttggctcttatccttgtaacgatgacttgtccctgcagatgcttggcatgcacgggactgtgtatgctaactacgctgtggagcatagtgatttgttgctggcgtttggtgttaggtttgatgaccgtgtcacgggaaagctcgaggcgtttgcgagcagggctaagattgtgcacatagacattgattctgctgagattgggaagaataagacacctcacgtgtctgtgtgtggtgatgtaaagctggctttgcaagggatgaacaaggttcttgagaaccgggcggaggagctcaagcttgatttcggtgtttggaggagtgagttgagcgagcagaaacagaagttcccgttgagcttcaaaacgtttggagaagccattcctccgcagtacgcgattcaggtcctagacgagctaacccaagggaaggcaattatcagtactggtgttggacagcatcagatgtgggcggcgcagttttacaagtacaggaagccgaggcagtggctgtcgtcctcaggactcggagctatgggtttcggacttcctgctgcgattggagcgtctgtggcgaaccctgatgcgattgttgtggacattgacggtgatggaagcttcataatgaacgttcaagagctggccacaatccgtgtagagaatcttcctgtgaagatactcttgttaaacaaccagcatcttgggatggtcatgcaatgggaagatcggttctacaaagctaacagagctcacacttatctcggggacccggcaagggagaacgagatcttccctaacatgctgcagtttgcaggagcttgcgggattccagctgcgagagtgacgaagaaagaagaactccgagaagctattcagacaatgctggatacacctggaccgtacctgttggatgtcatctgtccgcaccaagaacatgtgttaccgatgatcccaagtggtggcactttcaaagatgtaataaccgaaggggatggtcgcactaagtactga

>***BnALS3-Zheyou51***

atggcggcggcaacatcgtcttctCCGATCTCCTTAACCGCTAAACCTTCTTCCAAATCCCCTCTACCCATTTCCAGATT

CTCCCTTCCCTTCTCCTTAACCCCACAGAAACCCTCCTCCCGTCTCCACCGTCCACTCGCCATCTCCGCCGTTCTCAACTCACCCGTCAATGTCGCACCTGAAAAAACCGACAAGATCAAGACTTTCATCTCCCGCTACGCTCCCGACGAGCCCCGCAAGGGTGCTGATATCCTCGTGGAAGCCCTCGAGCGTCAAGGCGTCGAAACCGTCTTCGCTTATCCCGGAGGTGCTTCCATGGAGATCCACCAAGCCTTGACTCGCTCCTCCACCATCCGTAACGTCCTTCCCCGTCACGAACAAGGAGGAGTCTTCGCCGCCGAGGGTTACGCTCGTTCCTCCGGCAAACCGGGAATCTGCATAGCCACTTCGGGTCCCGGAGCTACCAACCTCGTCAGCGGGTTAGCAGACGCGATGCTTGACAGTGTTCCTCTCGTCGCCATCACAGGACAGGTCCCTCGCCGGATGATCGGTACTGACGCGTTCCAAGAGACGCCAATCGTTGAGGTAACGAGGTCTATTACGAAACATAACTATCTGGTGATGGATGTTGATGACATACCTAGGATCGTTCAAGAAGCTTTCTTTCTAGCTACTTCCGGTAGACCCGGACCGGTTTTGGTTGATGTTCCTAAGGATATTCAGCAGCAGCTTGCGATTCCTAACTGGGATCAACCTATGCGCTTGCCTGGCTACATGTCTAGGCTGCCTCAGCCACCGGAAGTTTCTCAGTTAGGCCAGATCGTTAGGTTGATCTCGGAGTCTAAGAGGCCTGTTTTGTACGTTGGTGGTGGAAGCTTGAACTCGAGTGAAGAACTGGGGAGATTTGTCGAGCTTACTGGGATCCCCGTTGCGAGTACGTTGATGGGGCTTGGCTCTTATCCTTGTAACGATGAGTTGTCCCTGCAGATGCTTGGCATGCACGGGACTGTGTATGCTAACTACGCTGTGGAGCATAGTGATTTGTTGCTGGCGTTTGGTGTTAGGTTTGATGACCGTGTCACGGGAAAGCTCGAGGCGTTTGCGAGCAGGGCTAAGATTGTGCACATAGACATTGATTCTGCTGAGATTGGGAAGAATAAGACACCTCACGTGTCTGTGTGTGGTGATGTAAAGCTGGCTTTGCAAGGGATGAACAAGGTTCTTGAGAACCGGGCGGAGGAGCTCAAGCTTGATTTCGGTGTTTGGAGGAGTGAGTTGAGCGAGCAGAAACAGAAGTTCCCGTTGAGCTTCAAAACGTTTGGAGAAGCCATTCCTCCGCAGTACGCGATTCAGGTCCTAGACGAGCTAACCCAAGGGAAGGCAATTATCAGTACTGGTGTTGGACAGCATCAGATGTGGGCGGCGCAGTTTTACAAGTACAGGAAGCCGAGGCAGTGGCTGTCGTCCTCAGGACTCGGAGCTATGGGTTTCGGACTTCCTGCTGCGATTGGAGCGTCTGTGGCGAACCCTGATGCGATTGTTGTGGACATTGACGGTGATGGAAGCTTCATAATGAACGTTCAAGAGCTGGCCACAATCCGTGTAGAGAATCTTCCTGTGAAGATACTCTTGTTAAACAACCAGCATCTTGGGATGGTCATGCAATGGGAAGATCGGTTCTACAAAGCTAACAGAGCTCACACTTATCTCGGGGACCCGGCAAGGGAGAACGAGATCTTCCCTAACATGCTGCAGTTTGCAGGAGCTTGCGGGATTCCAGCTGCGAGAGTGACGAAGAAAGAAGAACTCCGAGAAGCTATTCAGACAATGCTGGATACACCTGGACCGTACCTGTTGGATGTCATCTGTCCGCACCAAGAACATGTGTTACCGATGATCCCAAGTGGTGGCACTTTCAAAGATGTAATAACCGAAGGGGATGGTCGCACTAAGTACTGA

>***BnALS3-Zheshuang72***

atggcggcggcaacatcgtcttctCCGATCTCCTTAACCGCTAAACCTTCTTCCAAATCCCCTCTACCCATTTCCAGATT

CTCCCTTCCCTTCTCCTTAACCCCACAGAAACCCTCCTCCCGTCTCCACCGTCCACTCGCCATCTCCGCCGTTCTCAACTCACCCGTCAATGTCGCACCTGAAAAAACCGACAAGATCAAGACTTTCATCTCCCGCTACGCTCCCGACGAGCCCCGCAAGGGTGCTGATATCCTCGTGGAAGCCCTCGAGCGTCAAGGCGTCGAAACCGTCTTCGCTTATCCCGGAGGTGCTTCCATGGAGATCCACCAAGCCTTGACTCGCTCCTCCACCATCCGTAACGTCCTTCCCCGTCACGAACAAGGAGGAGTCTTCGCCGCCGAGGGTTACGCTCGTTCCTCCGGCAAACCGGGAATCTGCATAGCCACTTCGGGTCCCGGAGCTACCAACCTCGTCAGCGGGTTAGCAGACGCGATGCTTGACAGTGTTCCTCTCGTCGCCATCACAGGACAGGTCCCTCGCCGGATGATCGGTACTGACGCGTTCCAAGAGACGCCAATCGTTGAGGTAACGAGGTCTATTACGAAACATAACTATCTGGTGATGGATGTTGATGACATACCTAGGATCGTTCAAGAAGCTTTCTTTCTAGCTACTTCCGGTAGACCCGGACCGGTTTTGGTTGATGTTCCTAAGGATATTCAGCAGCAGCTTGCGATTCCTAACTGGGATCAACCTATGCGCTTGCCTGGCTACATGTCTAGGCTGCCTCAGCCACCGGAAGTTTCTCAGTTAGGCCAGATCGTTAGGTTGATCTCGGAGTCTAAGAGGCCTGTTTTGTACGTTGGTGGTGGAAGCTTGAACTCGAGTGAAGAACTGGGGAGATTTGTCGAGCTTACTGGGATCCCCGTTGCGAGTACGTTGATGGGGCTTGGCTCTTATCCTTGTAACGATGAGTTGTCCCTGCAGATGCTTGGCATGCACGGGACTGTGTATGCTAACTACGCTGTGGAGCATAGTGATTTGTTGCTGGCGTTTGGTGTTAGGTTTGATGACCGTGTCACGGGAAAGCTCGAGGCGTTCGCTAGCAGGGCTAAGATTGTGCACATAGACATTGATTCTGCTGAGATTGGGAAGAATAAGACACCTCACGTGTCTGTGTGTGGTGATGTAAAGCTGGCTTTGCAAGGGATGAACAAGGTTCTTGAGAACCGGGCGGAGGAGCTCAAGCTTGATTTCGGTGTTTGGAGGAGTGAGTTGAGCGAGCAGAAACAGAAGTTCCCGTTGAGCTTCAAAACGTTTGGAGAAGCCATTCCTCCGCAGTACGCGATTCAGGTCCTAGACGAGCTAACCCAAGGGAAGGCAATTATCAGTACTGGTGTTGGACAGCATCAGATGTGGGCGGCGCAGTTTTACAAGTACAGGAAGCCGAGGCAGTGGCTGTCGTCCTCAGGACTCGGAGCTATGGGTTTCGGACTTCCTGCTGCGATTGGAGCGTCTGTGGCGAACCCTGATGCGATTGTTGTGGACATTGACGGTGATGGAAGCTTCATAATGAACGTTCAAGAGCTGGCCACAATCCGTGTAGAGAATCTTCCTGTGAAGATACTCTTGTTAAACAACCAGCATCTTGGGATGGTCATGCAATGGGAAGATCGGTTCTACAAAGCTAACAGAGCTCACACTTATCTCGGGGACCCGGCAAGGGAGAACGAGATCTTCCCTAACATGCTGCAGTTTGCAGGAGCTTGCGGGATTCCAGCTGCGAGAGTGACGAAGAAAGAAGAACTCCGAGAAGCTATTCAGACAATGCTGGATACACCTGGACCGTACCTGTTGGATGTCATCTGTCCGCACCAAGAACATGTGTTACCGATGATCCCAAGTGGTGGCACTTTCAAAGATGTAATAACCGAAGGGGATGGTCGCACTAAGTACTGA

>***BnALS3-5N***

atggcggcggcaacatcgtcttctCCGATCTCCTTAACCGCTAAACCTTCTTCCAAATCCCCTCTACCCATTTCCAGATT

CTCCCTTCCCTTCTCCTTAACCCCACAGAAACCCTCCTCCCGTCTCCACCGTCCACTCGCCATCTCCGCCGTTCTCAACTCACCCGTCAATGTCGCACCTGAAAAAACCGACAAGATCAAGACTTTCATCTCCCGCTACGCTCCCGACGAGCCCCGCAAGGGTGCTGATATCCTCGTGGAAGCCCTCGAGCGTCAAGGCGTCGAAACCGTCTTCGCTTATCCCGGAGGTGCCTCCATGGAGATCCACCAAGCCTTGACTCGCTCCTCCACCATCCGTAACGTCCTCCCCCGTCACGAACAAGGAGGAGTCTTCGCCGCCGAGGGTTACGCTCGTTCCTCCGGCAAACCGGGAATCTGCATAGCCACTTCGGGTCCCGGAGCTACCAACCTCGTCAGCGGGTTAGCCGACGCGATGCTTGACAGTGTTCCTCTCGTCGCCATCACAGGACAGGTCCCTCGCCGGATGATCGGTACTGACGCGTTCCAAGAGACGCCAATCGTTGAGGTAACGAGGTCTATTACGAAACATAACTATCTGGTGATGGATGTTGATGACATACCTAGGATCGTTCAAGAAGCATTCTTTCTAGCTACTTCCGGTAGACCCGGACCGGTTTTGGTTGATGTTCCTAAGGATATTCAGCAGCAGCTTGCGATTCCTAACTGGGATCAACCTATGCGCTTGCCTGGCTACATGTCTAGGCTGCCTCAGCCACCGGAAGTTTCTCAGTTAGGtCAGATCGTTAGGTTGATCTCGGAGTCTAAGAGGCCTGTTTTGTACGTTGGTGGTGGAAGCTTGAACTCGAGTGAAGAACTGGGGAGATTTGTCGAGCTTACTGGGATCCCTGTTGCGAGTACGTTGATGGGGCTTGGCTCTTATCCTTGTAACGATGAGTTGTCCCTGCAGATGCTTGGCATGCACGGGACTGTGTATGCTAACTACGCTGTGGAGCATAGTGATTTGTTGCTGGCGTTTGGTGTTAGGTTTGATGACCGTGTCACGGGAAAGCTCGAGGCGTTTGCGAGCAGGGCTAAGATTGTGCACATAGACATTGATTCTGCTGAGATTGGGAAGAATAAGACACCTCACGTGTCTGTGTGTGGTGATGTAAAGCTGGCTTTGCAAGGGATGAACAAGGTTCTTGAGAACCGGGCGGAGGAGCTCAAGCTTGATTTCGGTGTTTGGAGGAGTGAGTTGAGCGAGCAGAAACAGAAGTTCCCGTTGAGCTTCAAAACGTTTGGAGAAGCCATTCCTCCGCAGTACGCGATTCAGGTCCTAGACGAGCTAACCCAAGGGAAGGCAATTATCAGTACTGGTGTTGGACAGCATCAGATGTGGGCGGCGCAGTTTTACAAGTACAGGAAGCCGAGGCAGTGGCTGTCGTCCTCAGGACTCGGAGCTATGGGTTTCGGACTTCCTGCTGCGATTGGAGCGTCTGTGGCGAACCCTGATGCGATTGTTGTGGACATTGACGGTGATGGAAGCTTCATAATGAACGTTCAAGAGCTGGCCACAATCCGTGTAGAGAATCTTCCTGTGAAGATACTCTTGTTAAACAACCAGCATCTTGGGATGGTCATGCAATTGGAAGATCGGTTCTACAAAGCTAACAGAGCTCACACTTATCTCGGGGACCCGGCAAGGGAGAACGAGATCTTCCCTAACATGCTGCAGTTTGCAGGAGCTTGCGGGATTCCAGCTGCGAGAGTGACGAAGAAAGAAGAACTCCGAGAAGCTATTCAGACAATGCTGGATACACCTGGACCGTACCTGTTGGATGTCATCTGTCCGCACCAAGAACATGTGTTACCGATGATCCCAAGTGGTGGCACTTTCAAAGATGTAATAACCGAAGGGGATGGTCGCACTAAGTACTGA
