# Supplementary material for: Development and validation of functional kompetitive allele-specific PCR markers for herbicide resistance in Brassica napus
Source: Front Plant Sci. 2023 Nov 23;14:1213476. doi: 10.3389/fpls.2023.1213476 (PMC10701909; doi:10.3389/fpls.2023.1213476)
Supplement: Supplementary file 2 [file DataSheet_2.docx]

>**AtALS**

MAAATTTTTTSSSISFSTKPSPSSSKSPLPISRFSLPFSLNPNKSSSSSRRRGIKSSSPSSISAVLNTTTNVTTTPSPTK

PTKPETFISRFAPDQPRKGADILVEALERQGVETVFAYPGGASMEIHQALTRSSSIRNVLPRHEQGGVFAAEGYARSSGKPGICIATSGPGATNLVSGLADALLDSVPLVAITGQVPRRMIGTDAFQETPIVEVTRSITKHNYLVMDVEDIPRIIEEAFFLATSGRPGPVLVDVPKDIQQQLAIPNWEQAMRLPGYMSRMPKPPEDSHLEQIVRLISESKKPVLYVGGGCLNSSDELGRFVELTGIPVASTLMGLGSYPCDDELSLHMLGMHGTVYANYAVEHSDLLLAFGVRFDDRVTGKLEAFASRAKIVHIDIDSAEIGKNKTPHVSVCGDVKLALQGMNKVLENRAEELKLDFGVWRNELNVQKQKFPLSFKTFGEAIPPQYAIKVLDELTDGKAIISTGVGQHQMWAAQFYNYKKPRQWLSSGGLGAMGFGLPAAIGASVANPDAIVVDIDGDGSFIMNVQELATIRVENLPVKVLLLNNQHLGMVMQWEDRFYKANRAHTFLGDPAQEDEIFPNMLLFAAACGIPAARVTKKADLREAIQTMLDTPGPYLLDVICPHQEHVLPMIPSGGTFNDVITEGDGRIKY.

>**BnALS1-Zheyou50**

MAAATSSSPISLTAKPSSKSPLPISRFSLPFSLTPQKDSSRLHRPLAISAVLNSPVNVAPPSPEKTDKNKTFVSRYAPDEPRKGADILVEALERQGVETVFAYPGGASMEIHQALTRSSTIRNVLPRHEQGGVFAAEGYARSSGKPGICIATSGPGATNLVSGLADAMLDSVPLVAITGQVPRRMIGTDAFQETPIVEVTRPVLYVGGGSLNSSEELGRFVELTGIPVASTLMGLGSYPCNDELSLQMLGMHGTVYANYAVEHSDLLLAFGVRFDDRVTGKLEAFASRAKIVHIDIDSAEIGKNKTPHVSVCGDVKLALQGMNKVLENRAEELKLDFGVWRSELSEQKQKFPLSFKTFGEAIPPQYAIQILDELTEGKAIISTGVGQHQMWAAQFYKYRKPRQWLSSSGLGAMGFGLPAAIGASVANPDAIVVDIDGDGSFIMNVQELATIRVENLPVKILLLNNQHLGMVMQWEDRFYKANRAHTYLGDPARENEIFPNMLQFAGACGIPAARVTKKEELREAIQTMLDTPGPYLLDVICPHQEHVLPMIPSGGTFKDVITEGDGRTKY.

>**BnALS1-Zheyou51**

MAAATSSSPISLTAKPSSKSPLPISRFSLPFSLTPQKDSSRLHRPLAISAVLNSPVNVAPPSPEKTDKNKTFVSRYAPDEPRKGADILVEALERQGVETVFAYPGGASMEIHQALTRSSTIRNVLPRHEQGGVFAAEGYARSSGKPGICIATSGPGATNLVSGLADAMLDSVPLVAITGQVPRRMIGTDAFQETPIVEVTRPVLYVGGGSLNSSEELGRFVELTGIPVASTLMGLGSYPCNDELSLQMLGMHGTVYANYAVEHSDLLLAFGVRFDDRVTGKLEAFASRAKIVHIDIDSAEIGKNKTPHVSVCGDVKLALQGMNKVLENRAEELKLDFGVWRSELSEQKQKFPLSFKTFGEAIPPQYAIQILDELTEGKAIISTGVGQHQMWAAQFYKYRKPRQWLSSSGLGAMGFGLPAAIGASVANPDAIVVDIDGDGSFIMNVQELATIRVENLPVKILLLNNQHLGMVMQWEDRFYKANRAHTYLGDPARENEIFPNMLQFAGACGIPAARVTKKEELREAIQTMLDTPGPYLLDVICPHQEHVLPMIPSGGTFKDVITEGDGRTKY.

>**BnALS1-Zheshuang72**

MAAATSSSPISLTAKPSSKSPLPISRFSLPFSLTPQKDSSRLHRPLAISAVLNSPVNVAPPSPEKIDKNKTFVSRYAPDEPRKGADILVEALERQGVETVFAYPGGASMEIHQALTRSSTIRNVLPRHEQGGVFAAEGYARSSGKPGICIATSGPGATNLVSGLADAMLDSVPLVAITGQVPRRMIGTDAFQETPIVEVTRPVLYVGGGSLNSSEELGRFVELTGIPVASTLMGLGSYPCNDELSLQMLGMHGTVYANYAVEHSDLLLAFGVRFDDRVTGKLEAFASRAKIVHIDIDSAEIGKNKTPHVSVCGDVKLALQGMNKVLENRAEELKLDFGVWRSELSEQKQKFPLSFKTFGEAIPPQYAIQILDELTEGKAIISTGVGQHQMWAAQFYKYRKPRQWLSSSGLGAMGFGLPAAIGASVANPDAIVVDIDGDGSFIMNVQELATIRVENLPVKILLLNNQHLGMVMQWEDRFYKANRAHTYLGDPARENEIFPNMLQFAGACGIPAARVTKKEELREAIQTMLDTPGPYLLDVICPHQEHVLPMIPSGGTFKDVITEGDGRTKY.

>**BnALS1-5N**

MAAATSSSPISLTAKPSSKSPLPISRFSLPFSLTPQKDSSRLHRPLAISAVLNSPVNVAPPSPEKTDKNKTFVSRYAPDEPRKGADILVEALERQGVETVFAYPGGASMEIHQALTRSSTIRNVLPRHEQGGVFAAEGYARSSGKPGICIATSGPGATNLVSGLADAMLDSVPLVAITGQVPRRMIGTDAFQETPIVEVTRPVLYVGGGSLNSSEELGRFVELTGIPVASTLMGLGSYPCNDELSLQMLGMHGTVYANYAVEHSDLLLAFGVRFDDRVTGKLEAFASRAKIVHIDIDSAEIGKNKTPHVSVCGDVKLALQGMNKVLENRAEELKLDFGVWRSELSEQKQKFPLSFKTFGEAIPPQYAIQILDELTEGKAIISTGVGQHQMWAAQFYKYRKPRQWLSSSGLGAMGFGLPAAIGASVANPDAIVVDIDGDGSFIMNVQELATIRVENLPVKILLLNNQHLGMVMQLEDRFYKANRAHTYLGDPARENEIFPNMLQFAGACGIPAARVTKKEELREAIQTMLDTPGPYLLDVICPHQEHVLPMIPSGGTFKDVITEGDGRTKY.

>**BnALS3-Zheyou50**

MAAATSSSPISLTAKPSSKSPLPISRFSLPFSLTPQKPSSRLHRPLAISAVLNSPVNVAPEKTDKIKTFISRYAPDEPRK

GADILVEALERQGVETVFAYPGGASMEIHQALTRSSTIRNVLPRHEQGGVFAAEGYARSSGKPGICIATSGPGATNLVSGLADAMLDSVPLVAITGQVPRRMIGTDAFQETPIVEVTRPVLYVGGGSLNSSEELGRFVELTGIPVASTLMGLGSYPCNDDLSLQMLGMHGTVYANYAVEHSDLLLAFGVRFDDRVTGKLEAFASRAKIVHIDIDSAEIGKNKTPHVSVCGDVKLALQGMNKVLENRAEELKLDFGVWRSELSEQKQKFPLSFKTFGEAIPPQYAIQVLDELTQGKAIISTGVGQHQMWAAQFYKYRKPRQWLSSSGLGAMGFGLPAAIGASVANPDAIVVDIDGDGSFIMNVQELATIRVENLPVKILLLNNQHLGMVMQWEDRFYKANRAHTYLGDPARENEIFPNMLQFAGACGIPAARVTKKEELREAIQTMLDTPGPYLLDVICPHQEHVLPMIPSGGTFKDVITEGDGRTKY.

>**BnALS3-Zheyou51**

MAAATSSSPISLTAKPSSKSPLPISRFSLPFSLTPQKPSSRLHRPLAISAVLNSPVNVAPEKTDKIKTFISRYAPDEPRK

GADILVEALERQGVETVFAYPGGASMEIHQALTRSSTIRNVLPRHEQGGVFAAEGYARSSGKPGICIATSGPGATNLVSGLADAMLDSVPLVAITGQVPRRMIGTDAFQETPIVEVTRPVLYVGGGSLNSSEELGRFVELTGIPVASTLMGLGSYPCNDELSLQMLGMHGTVYANYAVEHSDLLLAFGVRFDDRVTGKLEAFASRAKIVHIDIDSAEIGKNKTPHVSVCGDVKLALQGMNKVLENRAEELKLDFGVWRSELSEQKQKFPLSFKTFGEAIPPQYAIQVLDELTQGKAIISTGVGQHQMWAAQFYKYRKPRQWLSSSGLGAMGFGLPAAIGASVANPDAIVVDIDGDGSFIMNVQELATIRVENLPVKILLLNNQHLGMVMQWEDRFYKANRAHTYLGDPARENEIFPNMLQFAGACGIPAARVTKKEELREAIQTMLDTPGPYLLDVICPHQEHVLPMIPSGGTFKDVITEGDGRTKY.

>**BnALS3-Zheshuang72**

MAAATSSSPISLTAKPSSKSPLPISRFSLPFSLTPQKPSSRLHRPLAISAVLNSPVNVAPEKTDKIKTFISRYAPDEPRK

GADILVEALERQGVETVFAYPGGASMEIHQALTRSSTIRNVLPRHEQGGVFAAEGYARSSGKPGICIATSGPGATNLVSGLADAMLDSVPLVAITGQVPRRMIGTDAFQETPIVEVTRPVLYVGGGSLNSSEELGRFVELTGIPVASTLMGLGSYPCNDELSLQMLGMHGTVYANYAVEHSDLLLAFGVRFDDRVTGKLEAFASRAKIVHIDIDSAEIGKNKTPHVSVCGDVKLALQGMNKVLENRAEELKLDFGVWRSELSEQKQKFPLSFKTFGEAIPPQYAIQVLDELTQGKAIISTGVGQHQMWAAQFYKYRKPRQWLSSSGLGAMGFGLPAAIGASVANPDAIVVDIDGDGSFIMNVQELATIRVENLPVKILLLNNQHLGMVMQWEDRFYKANRAHTYLGDPARENEIFPNMLQFAGACGIPAARVTKKEELREAIQTMLDTPGPYLLDVICPHQEHVLPMIPSGGTFKDVITEGDGRTKY.

>**BnALS3-5N**

MAAATSSSPISLTAKPSSKSPLPISRFSLPFSLTPQKPSSRLHRPLAISAVLNSPVNVAPEKTDKIKTFISRYAPDEPRK

GADILVEALERQGVETVFAYPGGASMEIHQALTRSSTIRNVLPRHEQGGVFAAEGYARSSGKPGICIATSGPGATNLVSGLADAMLDSVPLVAITGQVPRRMIGTDAFQETPIVEVTRPVLYVGGGSLNSSEELGRFVELTGIPVASTLMGLGSYPCNDELSLQMLGMHGTVYANYAVEHSDLLLAFGVRFDDRVTGKLEAFASRAKIVHIDIDSAEIGKNKTPHVSVCGDVKLALQGMNKVLENRAEELKLDFGVWRSELSEQKQKFPLSFKTFGEAIPPQYAIQVLDELTQGKAIISTGVGQHQMWAAQFYKYRKPRQWLSSSGLGAMGFGLPAAIGASVANPDAIVVDIDGDGSFIMNVQELATIRVENLPVKILLLNNQHLGMVMQLEDRFYKANRAHTYLGDPARENEIFPNMLQFAGACGIPAARVTKKEELREAIQTMLDTPGPYLLDVICPHQEHVLPMIPSGGTFKDVITEGDGRTKY.
