# Supplementary figures and images for: Development and validation of functional kompetitive allele-specific PCR markers for herbicide resistance in Brassica napus
Source: Front Plant Sci. 2023 Nov 23;14:1213476. doi: 10.3389/fpls.2023.1213476 (PMC10701909; doi:10.3389/fpls.2023.1213476)

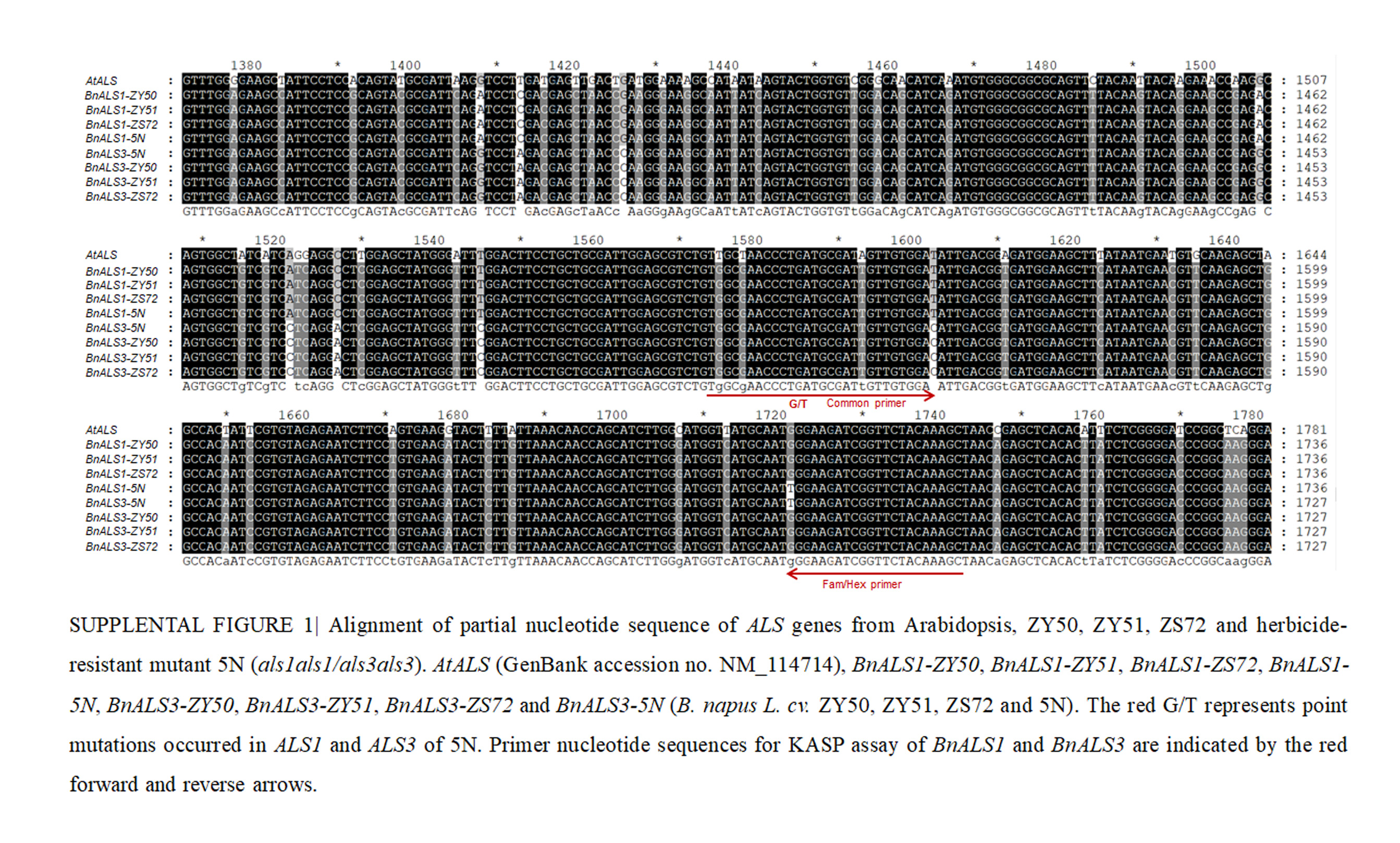

Supplement: Supplementary file 3 [file Image_1.jpeg]

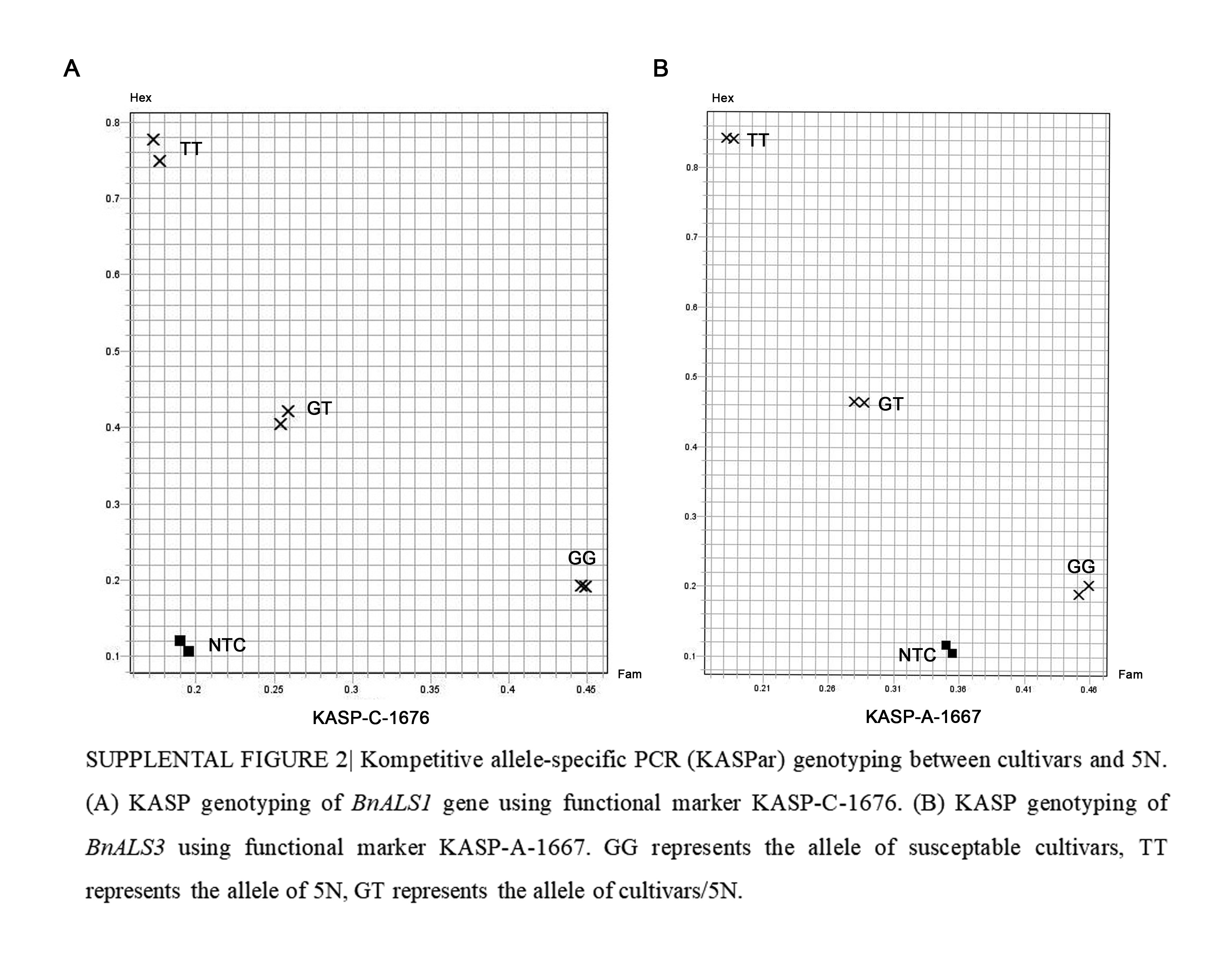

Supplement: Supplementary file 4 [file Image_2.jpeg]
